# The Dental Encyclopædia

**Published:** 1885-02

**Authors:** 


### THE DENTAL ENCYCLOPÆDIA.

PUBLISHED BY REQUEST OF THE SOCIETIES.

*To the Members of the Seventh and Eighth District Dental Societies of the State of New York:*

GENTLEMEN,

You did me the honor, in October, 1875, at your joint meeting in Buffalo, to invite me, by resolution of Dr. Line, to elaborate a paper on the need of a Dental Cyclopædia, and read it before the State Society at its next meeting, May, 1876. I did so.

Many of you, probably, read the report of the committee appointed to investigate and report on the subject in May, 1877, in the *Transactions* published in 1878, two years after. It is not my purpose to write of what was doing for the Encyclopædia from the time that report was read till a corrected proof of my paper was published in the May number, 1878, of the *Oral Science Magazine.* But it became very clear to the writer, as well as to others, who encouraged the project long before that report was published, that to ask any society to assume the pecuniary responsibility in publishing such a work was absurd ; but the idea that a great society might sanction and foster such a project seemed reasonable and possible.

But that was not seriously attempted, for it happened that in July, 1878, Dr. Buck, editor of the English edition of Ziemmssen's Encyclopædia of Medicine, was shown a copy of my article on the *Dental Cyclopœdia,* by a medical friend of his. Dr. Buck was so well pleased with the project that he was at some pains next morning to talk with Wm. Wood, of the publishing house of Wm. Wood & Co., of New York City, about it, with the following effect:

"New York, July 9, 1878.

"B. R. McGregor,

"*Dear Sir:*—Dr. Buck has spoken to us in regard to a plan of yours for a Cyclopædia of Dentistry. While we should hesitate to undertake any very heavy work just now, we should be glad to have your views concerning it at your leisure—number of vols? pages? authors? possible number of subscribers? etc.

"Very respectfully yours,

"WM. WOOD, & CO."

Mr. Wood was informed next day, in the concurrent words of those agitating the subject, that as soon as approximations of answers to his questions could be given, they would be made.

Here was a publishing capitalist, whose concern had just made $75,000 from publishing the English edition of Ziemmssen's Medical Encyclopædia, voluntarily offering to consider the subject of a Dental Encyclopædia, if it could be put into an intelligible form, and willing to put his capital into it and produce it, if he could but know what it was to be, etc.

The projectors of the work instantly set themselves with redoubled energy to develop, in the first place, a plan of the work. That was so far effected within a year as to be easily made presentable to Mr. Wood, and it would have more than answered his questions, (except that about the probable number of subscribers) but for obstacles that were not, if they could have been, overcome.

The writer well knows that the Odontological Society of New York would not commit itself to auspicate the work. Then there was a singular apathy and indifference to the project among dentists generally. He was constantly reminded of the caution he had received in a letter from the editor of the *Dental Cosmos*, months before, to expect nothing but failure in trying to interest the profession in it, and the words of others indicating that the profession was not ready for such a work. This is easily explained.

The people of England were not without cyclopædias before the first really successful one was produced by Chambers, in 1728. That work, however, was the supply of an unmistakable demand; and we know that it created new wants, since larger and better works followed from that time till now.

No, gentlemen, the project of the Dental Encyclopædia at that time was a mistake—worse, a blunder. It could not help but fail, and it did fail.

In a long and checkered career I have always received your unswerving kindness—often I have not deserved it;—certainly I have reason to thank you, gentlemen, and think you will believe me when I say that I part from you regretfully, but retain my interest in the profession, even more than before.

Very respectfully yours,

RUSH McGREGOR.

